# Mapping Socioecological Interconnections in One Health Across Human, Animal, and Environmental Health: A Scoping Review Protocol

**DOI:** 10.3390/ijerph23010098

**Published:** 2026-01-10

**Authors:** Jessica Farias Dantas Medeiros, Leonor Maria Pacheco Santos, Sindy Maciel Silva, Jorge Otávio Maia Barreto, Johnathan Portela da Silva Galdino, Eveline Fernandes Nascimento Vale, Kary Desiree Santos Mercedes, Mayara Suelirta da Costa, Juliana Michelotti Fleck, Karine Suene Mendes Almeida, Verônica Cortez Ginani, Wildo Navegantes de Araújo, Diule Vieira de Queiroz, Christina Pacheco

**Affiliations:** 1 Escola de Governo, Fundação Oswaldo Cruz, Brasília 70904-130, Brazil; fariasjessicad@gmail.com (J.F.D.M.); jorge.barreto@fiocruz.br (J.O.M.B.); johnathanportela@gmail.com (J.P.d.S.G.); mayarasuelirtac@gmail.com (M.S.d.C.); 2 Programa de Pós-Graduação em Saúde Coletiva, Universidade de Brasília, Brasília 70910-900, Brazil; valeeveline77@gmail.com (E.F.N.V.); karysantos017@gmail.com (K.D.S.M.); vcginani@unb.br (V.C.G.); wildo@unb.br (W.N.d.A.); 3 Global Health and Tropical Medicine, Instituto de Higiene e Medicina Tropical, Universidade Nova de Lisboa, Lisboa 1349-008, Portugal; sindy.silva@ihmt.unl.pt; 4 Fundação de Apoio à Fiocruz, Fiotec, Brasília 70904-130, Brazil; julianafleck@gmail.com; 5 Programa de Pós-Graduação em Medicina Tropical, Universidade de Brasília, Brasília 70910-900, Brazil; karine.suene@gmail.com; 6 Biblioteca Central, Universidade de Brasília, Brasília 70910-900, Brazil; diule@unb.br; 7 Programa de Pós-Graduação Multicêntrico em Ciências Fisiológicas, Universidade Federal da Paraíba, João Pessoa 58051-900, Brazil; christinaosvaldo@yahoo.com.br

**Keywords:** one health, food safety, zoonotic diseases, antimicrobial resistance, environmental health, scoping review protocol

## Abstract

The One Health framework highlights the interconnectedness of human, animal, and environmental health, requiring interdisciplinary and multisectoral collaboration to address complex global health challenges. This scoping review protocol aims to guide the systematic mapping on how studies and policy initiatives have incorporated socioecological interconnections within the One Health paradigm, following the Joanna Briggs Institute guidance and the PRISMA Scr checklist. The experimental design includes searches in PubMed, Scopus, Web of Science, LILACS, Health Systems Evidence, Social Systems Evidence, and Google Scholar for the period from 2004 to 2025. The strategy, developed with librarian support and peer reviewed, includes terms in English, Portuguese, and Spanish. Pilot searches retrieved 5333 PubMed and 470 LILACS records. Eligible documents must explicitly present two or more of the six One Health dimensions: policies to strengthen health systems; antimicrobial resistance; food safety; environmental health; emerging and re-emerging zoonotic epidemics and pandemics; endemic zoonotic, neglected tropical and vector-borne diseases. A standardized tool was developed for data extraction, synthesizing in narrative, tabular, and graphical formats. The protocol’s utilization will provide comprehensive mapping of practices and policies, identifying achievements, barriers, and knowledge gaps to inform future strategies and strengthen global health governance.

## 1. Introduction

One Health is a unified, cooperative, transdisciplinary, and interconnected approach to human, animal, and environmental health, aiming to address the challenges posed by emerging and re-emerging diseases, pandemics, zoonoses, antimicrobial resistance, food safety, climate change, and other public health threats [1,2,3]. Early ideas linking the environment to health date back to the 1800’s with Hayes and Virchow. Much later Schwabe formulated the “One Medicine” concept in the 1970s, but effectively, One Health only consolidated as a formal global strategy in the 21st century [4,5,6,7].

A key milestone was the Wildlife Conservation Society’s 2004 report [8], followed by the tripartite alliance between the World Health Organization (WHO), the World Organization for Animal Health (WOAH), and the Food and Agriculture Organization (FAO) in 2010, which recognized shared responsibilities at the human–animal–environment interface [4]. The COVID-19 pandemic demonstrated how the local emergence of a lethal pathogen, likely of animal origin, can rapidly disrupt global public health [9], reinforcing the urgency of coordinated action. In 2022, the worsening climate crisis and the lessons learned from the pandemic led to the formal inclusion of the United Nations Environment Programme (UNEP), strengthening the current quadripartite alliance (WHO, WOAH, FAO, and UNEP), supported by an independent One Health High-Level Expert Panel (OHHLEP) [10].

Furthermore, recent initiatives, such as the Lancet One Health Commission [11], first convened in 2019, emphasized that the socioecological interconnections among humans, animals, and the environment form the foundation of One Health [11]. The Commission noted that while early One Health efforts primarily focused exclusively on zoonotic diseases, recent progress has expanded initiatives but also raised concerns about fragmentation and insufficient conceptual clarity [11]. Another conceptual publication on One Health in 2024 further defined the One Health six dimensions as: One Health policies to strengthen health systems; antimicrobial resistance; food safety; environmental health; emerging and re-emerging zoonotic epidemics and pandemics; endemic zoonotic, neglected tropical and vector-borne diseases, which remain the central focus of attention of One Health worldwide [1].

In this protocol, socioecological interconnection serves as the guiding criterion for data extraction and narrative synthesis across six dimensions: policy, antimicrobial resistance, food safety, zoonotic epidemics and endemic diseases. This interconnection is defined as the simultaneous and interdependent presence of two or more One Health dimensions as reflected in studies, policies, or practices. It encompasses the formulation and implementation of interdisciplinary methodologies, the co-production of evidence, and the explicit consideration of their interdisciplinary impacts and outcomes, including how actions in one dimension affect or depend on others.

Despite institutional and conceptual advances and the establishment of multiple international One Health networks over the past decade [12], the scientific literature still lacks a review that systematically examines the formulation and implementation of policies and practices addressing socioecological interconnections on a global scale. Preliminary searches in PROSPERO, Cochrane Library, Open Science Framework (OSF), and JBI Evidence Synthesis identified reviews focused on specific aspects: governance (2000–2024) [13], multisectoral collaborations (2018–2024) [14], epidemiological interventions (2024) [15], and regional analyses, such as the One Health triad in public health in Latin America (2016) [16]. Other reviews, such as the bibliometric review published in 2022 [17], highlight data gaps and the persistence of fragmented One Health frameworks. None, however, encompass the socioecological interconnections of the four core dimensions of One Health from a global, multilingual, and broadly scientific perspective.

Despite significant institutional progress, many One Health initiatives remain fragmented, addressing each dimension in isolation. In this context, the present protocol proposes an innovative *scoping review* designed to map, systematize, and analyze the socioecological interconnections across One Health dimensions, encompassing scientific evidence and gray literature published between January 2004 and March 2025 in English, Spanish, and Portuguese. Describing and understanding these interconnections is essential, as the One Health dimensions are inherently interdependent: actions in one domain [10], such as antimicrobial resistance control, directly influence others, including food safety and environmental health.

Understanding these socioecological interconnections is therefore essential to design policies and interventions that reflect the complexity of real-world health systems. Therefore, this protocol seeks to fill a conceptual and methodological gap by providing a tool to analyze the evolution of interconnected One Health approaches over the past two decades, identify emerging challenges and research priorities, and strengthen both the theoretical underpinnings and the practical implementation of the One Health paradigm at the global level.

### Research Questions and Aims

The main research questions of this scoping review protocol are the following:

- How have One Health studies addressed the socioecological interconnections of human, animal, and environmental health, particularly across food safety, zoonotic diseases, antimicrobial resistance, and environmental health?
- What do their methodologies, scope, and interdisciplinary collaborations reveal about key achievements, gaps, and future research priorities?

This scoping review protocol aims to map the scientific evidence and gray literature addressing the socioecological interconnections of One Health on a global scale, with emphasis on the food safety, zoonotic disease, antimicrobial resistance, and environmental health dimensions, to identify advancements, challenges, and future priorities. In order to achieve the main objective, the following specific goals were established:

- Identify and characterize studies, policies, and practices that simultaneously address two or more of the One Health dimensions cited above, analyzing their methodologies, scope, and levels of interdisciplinary connection.
- Examine how these initiatives have operationalized socio-ecological interconnection, including intersectoral coordination, evidence co-production, interdisciplinary methodologies, and reported impacts across dimensions.
- Systematize the advancements, results, and contributions achieved in implementing the interconnected One Health approach, highlighting experiences in different local, regional, and global contexts.
- Identify barriers, challenges, and knowledge gaps that limit the consolidation of the One Health socioecological approach.
- Propose recommendations for future research, policy formulation, and intersectoral practices, contributing to strengthening interdisciplinary collaboration and addressing global health threats.

This scoping review protocol followed the methodological framework developed by the Joanna Briggs Institute (JBI) [18] and the Preferred Reporting Items for Systematic Reviews and Meta-Analyses extension for Scoping Reviews (PRISMA-ScR) checklist [19]. 

## 2. Materials and Methods

### Eligibility Criteria

The mnemonic PCC (Participants, Concept, and Context) was adopted to structure the research question for the scoping review [18]. The PCC for this protocol is defined below and the reference concepts are expanded upon in Table 1:

- Participants—Publications of studies, policies, strategies, and regulations addressing the One Health approach,
- Concept—Socioecological interconnections of One Health, with an emphasis on the dimensions of One health policy, antimicrobial resistance, food safety, environmental health, emerging and re-emerging zoonotic epidemics/pandemics, endemic zoonotic, neglected tropical and vector-borne diseases.
- Context—Formulation and implementation of health policies and practices at a global level.

Inclusion criteria follows the PCC framework, incorporating original articles, formally established references, and publications that discuss strategies, experiences, and actions on the socioecological interconnections of One Health, published between 2004 and February 2025, and that directly and substantially address the One Health approach, even in a localized context.

The protocol also includes gray literature—dissertations and theses, reports from governmental and non-governmental organizations, policy documents, and other relevant sources—to ensure a comprehensive overview of the practices and strategies adopted. Letters to the Editor, conference abstracts, event proceedings, sponsored articles, incomplete articles, articles without results, preprints, or articles that were outside the topic of interest of the review been excluded. Additional records may be included after searches conducted in the publications’ references.

## 3. Experimental Design

### 3.1. Search Strategy

The search strategy was developed collaboratively by experts in the concept terms and a librarian, together with other stakeholders. The process included peer review to ensure consistency and methodological rigor. Initially, relevant terms, keywords, and synonyms for the topic were listed in the Medical Subject Headings (MeSH), followed by a pilot search in PubMed, focusing on the concepts of One Health, food safety, zoonotic diseases, antimicrobial resistance, and environmental health. Additional keywords were extracted from titles, abstracts, and indexed terms, ensuring the expansion of the vocabulary of the conceptual block in English, Spanish, and Portuguese (Appendix A, Table A1).

Pilot searches were performed in PubMed and Latin American and Caribbean Literature in Health Sciences (LILACS) via Virtual Health Library (BVS) to validate the combination of descriptors and keywords, employing Boolean operators (“AND”, “OR”) and cross-language synonyms. As presented in Table 2, the PubMed strategy retrieved 5333 records, and the multilingual LILACS strategy retrieved 470 records for the period January 2004 to March 2025. These pilot searches confirmed the adequacy and sensitivity of the conceptual blocks used.

As evidence sources, we propose the following scientific literature databases: Medical Literature Analysis and Retrieval System Online (MEDLINE) via PubMed (https://pubmed.ncbi.nlm.nih.gov/), Latin American and Caribbean Literature in Health Sciences (LILACS) via Virtual Health Library (BVS) (https://bvsalud.org), Health Systems Evidence (https://www.healthsystemsevidence.org/), Social Systems Evidence (https://www.socialsystemsevidence.org/), SciVerse Scopus (https://www.elsevier.com/products/scopus/search), Web of Science (https://clarivate.com/academia-government/scientific-and-academic-research/research-discovery-and-referencing/web-of-science/); all URL were accessed on 27 March 2025. The reference lists of the most relevant studies can also be subject to manual search.

The protocol recommends retrieving gray literature by a structured electronic search on Google Scholar (https://scholar.google.com), in “incognito mode”, thus avoiding the influence of the researcher’s browsing history, examining the first 100 documents retrieved.

### 3.2. Study Selection

This protocol outlines a scoping review with the following study selection guidelines. The studies identified from all proposed databases are fed into the Rayyan QCRI software, version 27 March 2025 [25], to remove duplicates. Afterward, the studies are evaluated and selected based on the eligibility criteria by two independent reviewers, following the double-blind methodology when reading the title and summary of the studies in the first phase. In case of disagreements, a third reviewer (master) is consulted for discussion and final decision, and then selected studies are input into Excel spreadsheets version 97-2003. In the second stage, the full text of the selected studies is read, and those excluded are justified according to previously established exclusion criteria.

### 3.3. Data Extraction and Analysis

A data extraction tool was prepared based on the model provided by JBI (Table 3). The tool includes detailed information about the studies, such as title, authors, year of publication, source, country, publication type, and language. Additionally, data will be collected on the study’s objective, the One Health approach addressed, and methodological characteristics, such as the methodology used, data collection methods, databases consulted, and data collection period.

After extraction, the data is then analyzed and summarized to meet the research objectives and organized into tables, charts, and narrative summaries, basing the analysis on a narrative synthesis, highlighting the main findings, uncertainties, the evidence’s applicability, strengths, limitations, and implications for managers and future research. Since this is a scoping review protocol, there is no critical assessment of the methodological quality or analysis of the risk of bias of the included studies [18]. The PRISMA-ScR guideline and the PRISMA Flow Diagram will structure and organize the review [26].

## 4. Expected Results

As the scoping review is currently underway, preliminary findings suggest that the protocol will provide a comprehensive global overview of how socioecological interconnections have been reported and operationalized across One Health initiatives over the past two decades. The ongoing review aims to document and analyze the efforts described in the literature and policy frameworks that seek to articulate food safety, zoonotic diseases, antimicrobial resistance, and environmental health within a unified approach. The study will systematize global experiences and strategies implemented under the One Health framework, identifying advances, gaps, and patterns in how these interconnections have been pursued. It will also highlight recurring challenges related to intersectoral coordination and the adaptation of policies and practices to local and regional realities.

Beyond mapping conceptual and practical trends, the review in progress seeks to contribute to more integrated and coordinated strategies among the human, animal, and environmental health sector. These outcomes are expected to support directly the global One Health agenda, and the Quadripartite Joint Plan of Action (2022–2026) [10]. This reinforces the originality and relevance of the study in advancing both the theoretical consolidation and the practical implementation of the One Health framework in global health governance.

By combining temporal and geographical perspectives, the ongoing review will trace the evolution of reported integration efforts across regions and health systems, revealing milestones, persistent gaps, and emerging priorities. Through its inclusive approach, without restrictions on geographic scope or language, and incorporating evidence published in Portuguese, English, and Spanish, the ongoing review expands the reach and representativeness of existing research. This comprehensive mapping will enable the identification of milestones, persistent gaps, and emerging priorities, providing robust evidence to guide sustainable, context-sensitive, and collaborative global health policies and interventions.

## Figures and Tables

**Table 1:** Definition of reference concepts for the scoping review Protocol’s PCC.

	Concept	Definition
Publication of studies, policies, strategies, and regulations addressing the One Health interface with health policies and practices	Policy	Peer-reviewed scientific studies, as well as institutional frameworks, guidelines, national or international plans, legislation, and governance structures, on prevention, promotion, evaluation and management addressing the One Health interface with health policies and practices [10].
Socioecological interconnections of One Health, with an emphasis on the dimensions of food safety, zoonotic diseases, antimicrobial resistance, and environmental health	Socioecological interconnections of One Health	The simultaneous and interdependent consideration of at least two dimensions of One Health: food safety, zoonotic diseases, antimicrobial resistance, and environmental health. The review will include publications that examine how these interconnections are formulated and implemented in policies, programs, interventions, and practices, as well as how they integrate methodologies, foster interdisciplinary collaboration, and report outcomes or challenges. Special attention will be given to initiatives that demonstrate explicit articulation of cross-sectoral or interdisciplinary impacts
	Antimicrobial resistance	Encompasses the emergence, spread, and control of resistance to antimicrobials (antibiotics, antivirals, antifungals, and antiparasitics) due to the excessive or inappropriate use of these drugs in humans, animals, and the environment [20].
	Food safety risks	Food safety, avoiding physical, chemical, and biological risks throughout the production chain (breeding, slaughter, processing, transportation, marketing) [21].
	Environmental health	Refers to the impacts of the environment on human and animal health, such as deforestation, pollution, inadequate waste management, land use, climate change, and biodiversity loss [22].
	Zoonotic diseases Epidemics Endemics	Zoonoses, or zoonotic diseases, are diseases transmitted between animals and humans, including those of viral, bacterial, or parasitic origin, which emerge especially in areas where wildlife, domestic animals, and humans interact [10,23]. Publications regarding zoonotic diseases will be further classified [24] as follows: EPI: emerging and re-emerging zoonotic epidemics and pandemics; END: endemic zoonotic, neglected tropical and vector-borne diseases.
Geographical location	Global level	No geographical restrictions applied. Publications will be considered across different regions, countries, and settings (local, regional, or international).

**Table 2:** Pilot search in the electronic database PubMed and LILACS.

Database	Key Words	Filter	Search Date	Number of Search Results
Pubmed	(“One Health” OR “One Medicine initiative”) AND (“Food safety” OR “Food quality” OR “Food contamination” OR Zoonoses OR Zoonosis OR “Zoonotic diseases” OR “Zoonotic Spillover” OR “Zoonotic Spillovers” OR “Microbial drug resistance” OR “Microbial antibiotic resistance” OR “Environmental health” OR “Environment and Public Health” OR “Climate Change”).	01/01/04 to 27/03/25	27/03/25	5333
LILACS	(“One Health” OR “One Medicine initiative” OR “Saúde Única” OR “Iniciativa Uma Só Medicina” OR “Iniciativa Uma Só Saúde” OR “Iniciativa Medicina Única” OR “concepto de Salud Única” OR “concepto de Una Sola Salud” OR “iniciativa de Una Sola Salud” OR “iniciativa una sola Medicina” OR “Salud Única”) AND (“Food safety” OR “Food contamination” OR “Food quality” OR “Microbial drug resistance” OR “Microbial antibiotic resistance” OR Zoonoses OR “Zoonotic diseases” OR “Zoonotic Spillover” OR “Zoonotic Spillovers” OR “Environmental health” OR “Environment and Public Health” OR “Climate Change” OR “Inocuidade dos Alimentos” OR “Contaminação de alimentos” OR “Qualidades de alimentos” OR “Resistência Microbiana a Antibióticos” OR “Resistência Microbiana a Drogas” OR “Resistência a Antibióticos” OR “doenças zoonóticas” OR “Derrame Zoonótico” OR “Saúde ambiental” OR “Saúde e Meio Ambiente” OR “Saúde pública ambiental” OR “Mudança Climática” OR “Alteração Climática” OR “Alterações Climáticas” OR “Inocuidad de los Alimentos” OR “Contaminación de Alimentos” OR “Calidad de los Alimentos” OR “Farmacorresistencia Microbiana” OR “resistencia microbiana a antibióticos” OR “resistencia microbiana a drogas” OR “resistencia microbiana a fármacos” OR “resistencia microbiana a medicamentos” OR “Enfermedades Zoonóticas” OR “Zoonosis” OR “Salud Ambiental” OR “Salud pública ambiental” OR “Salud y Ambiente” OR “alteración climática” OR “cambio climático”)	01/01/04 to 27/03/25	27/03/25	470

**Table 3:** Data extraction form.

Variable	Data to Be Collected
Key ID Rayyan	Internal code for study tracking
Full reference and web page	Title, year, periodical, volume, issue, pages, authors, web page
Publication type	Scientific paper; thesis; dissertation; editorial; protocol; technical document
Aims of the study	Objective of the practice or study, as informed by the authors of the article
Study context	Documental analysis; field study; laboratory study, as reported by the authors
Country and locality of the study	Country and/or locality where the practice or study was carried out, as informed by the authors
Data collection period	Data collection period (initial year and final year) reported in the article
Primary One Health dimension(s)	Main dimension of the article’s object of study: ONE: One Health Policies AMR: Antimicrobial Resistance FSA: Food Safety ENH: Environmental health ZOO/EPI: Emerging and re-emerging zoonotic epidemics and pandemics ZOO/END: Endemic zoonotic, neglected tropical and vector-borne diseases
Secondary One Health dimension(s)	Dimension which the article addressed in addition to the main dimension: ONE: One Health Policies AMR: Antimicrobial Resistance FSA: Food Safety ENH: Environmental health ZOO/EPI: emerging and re-emerging zoonotic epidemics and pandemics ZOO/END: endemic zoonotic, neglected tropical and vector-borne diseases
One Health socioecological interconnections	Direct and substantial presence of two or more dimensions of One Health in joint actions, methodologies, data or decisions, fostering intersectoral and transdisciplinary collaboration (e.g., how actions in one dimension affect or depend on others).
Transcript of a relevant direct quote from the One Health socioecological interconnections	Transcription of an excerpt from the interconnected One Health approach
Advancements, challenges, and relevant results for the One Health approach	Main results, challenges or advances obtained based on the intervention or practice analyzed
Additional information	Additional notes on personal impressions during the extraction process

## Data Availability

No new data were created or analyzed in this study. Data sharing is not applicable to this article.

## Appendix A

**Table A1:** Conceptual blocks and related terms.

Conceptual Blocks	Related Terms
One Health	One Health
	One Health concept
	One Health initiative
	One Medicine initiative
Food safety	Food safety
	food contamination
	food quality
Zoonotic diseases	Bacterial Zoonoses
	Viral Zoonoses
	Fungal zoonoses
	Protozoan Zoonoses
	Zoonoses
	Zoonotic Spillover
	Zoonotic Spillovers
	Zoonotic diseases
Antimicrobial resistance	Microbial drug resistance
	Microbial Antibiotic resistance
	Microbial drug resistance
Environmental Health	Environmental Health
	Health, Environmental
	Environment and Public Health
	Climate Change

Source: Prepared by the authors.
